# Cloning, Purification, and Characterization of a Heat- and Alkaline-Stable Endoglucanase B from *Aspergillus niger* BCRC31494

**DOI:** 10.3390/molecules17089774

**Published:** 2012-08-14

**Authors:** Chien-Huang Li, Hsing-Ren Wang, Tsong-Rong Yan

**Affiliations:** Department of Bioengineering, Tatung University, 40 Chung-Shang North Road, 3rd Section, Taipei 104, Taiwan; Email: ice200212@yahoo.com.tw (C.-H.L.); singren54@gmail.com (H.-R.W.)

**Keywords:** *Aspergillus niger*, endoglucanase B, gene cloning and expression, thermal and alkaline tolerance

## Abstract

Endoglucanase B (EGLB) derived from *Aspergillus niger* BCRC31494 has been used in the food fermentation industry because of its thermal and alkaline tolerance. It was cloned and expressed in *Pichia pastoris*. According to sequence analysis, the gene open reading frame comprises 1,217 bp with five introns (GenBank GQ292753). According to sequence and protein domain analyses, EGLB was assigned to glycosyl hydrolase family 5 of the cellulase superfamily. Several binding sites were found in the promoter region. The purified recombinant enzyme was induced by 0.5% methanol, and it exhibited optimal activity at 70 °C and pH 4. EGLB was stable for 3 h at temperatures below 60 °C, with more than 90% of its activity remaining. The enzyme was specific for substrates with β-1,3 and β-1,4 linkages. In Lineweaver-Burk plot analysis, the *K_m_* and *V_max_* values of EGLB for β-D-glucan were 134 mg/mL and 4.68 U/min/mg, respectively. The enzyme activity was increased by 1.86-fold by Co^2+^ and by 2-fold by Triton X-100 and Tween 80. These favorable properties make EGLB a potential candidate for use in laundry and textile industrial applications.

## 1. Introduction

Cellulose, a polymer of glucose with β-1,4 linkages, is the most abundant biomass on Earth. Its abundance and its renewability make it a great candidate energy source and feedstock. After several oil crises, bioethanol has been promoted as an alternative energy source, and the production of bioethanol from cellulosic biomass has been widely explored. Enzymes degrading cellulose are found in microbes, plants, and the digestive tracts of animals [1,2].

Cellulose is hydrolyzed by the synergistic reaction of cellulases. Cellulase has been used in the food, textile, detergent, and pulp and paper industries for several decades [3,4,5]. Another area in which cellulases have been extensively used recently is in the bioconversion of cellulosic material for bioethanol production [6]. The cellulase complex includes endoglucanases (EC 3.2.1.4), exoglucanases (EC 3.2.1.91), and β-glucosidases (EC 3.2.1.21). Endoglucanases randomly attack the internal chain of cellulose to produce cellooligosaccharides. Exoglucanases catalyze the hydrolysis of crystalline cellulose from the ends of the cellulose chain to produce cellobiose, which is ultimately hydrolyzed to glucose by β-glucosidases [7,8]. Glycoside hydrolases (GHs), including the aforementioned enzymes, have been classified into more than 130 families in the continually updated carbohydrate-active enzymes database (www.cazy.org). Endoglucanase belongs to 17 GH families, namely families 5, 6, 7, 8, 9, 12, 16, 17, 44, 45, 48, 51, 55, 61, 74, 81, and 124. Within GH5, which primarily contains endo-1,4-glucanases, six different subtypes (A1–A6) were proposed [8,9]. To utilize cellulose, various endoglucanases from fungi, bacteria, higher plants, and animals have been isolated, purified, and characterized [10]. Futhermore, microbial enzymes are economically important and environmentally friendly.

In fungi, several species, such as members of the genera *Trichoderma*, *Penicillium*, and *Aspergillus*, are widely used in industrial applications [11,12,13]. *Aspergillus niger* is the most important cellulase source for the fermentation of Asian foods [14]. This fungus can secrete large amounts of different cellulases, and it is recognized as one of the more efficient cellulose-degrading microorganisms. Endoglucanases are the enzymes that initiate the cellulose degradation pathway [8]. In previous reports, three endoglucanase genes, *egl*A, *egl*B, and *egl*C, had been found in *A. niger*. The full-length cDNA of *egl*A, *egl*B, and *egl*C were 720, 996, and 2,574 bp, respectively [15,16,17]. These endoglucanases have optimum temperature at 50 to 60 °C and optimum pH at 4 to 6. The molecular weight of *egl*A and *egl*B were 30 to 55 kDa and the two enzymes have high specificity toward beta-glucan. However, both two genes lack a cellulose-binding domain (CBD) and the associated linker region. The *egl*C had the large molecular weight about 90 kDa and exhibited greastest activity towards xyloglucan. Although no synergy was found towards eglA and eglB, eglC has a specific role in plant cell wall degradation [15,17,18]. In cellulose complex, a transcriptional activator XlnR regulates the transcription of the *xlnB*, *xlnC*, and *xlnD* genes encoding the main xylanolytic enzymes (endoxylanases B and C and β-xylosidase, respectively) [16]. The transcription of the genes encoding the accessory enzymes involved in xylan degradation, including α-glucuronidase A, acetylxylan esterase A, arabinoxylan arabinofuranohydrolase A, and feruloyl esterase A, was controlled by XlnR. The XlnR also activates transcription of three endoglucanase-encoding genes, *egl*A, *egl*B, and *egl*C, indicating that transcriptional regulation by XlnR goes beyond the genes encoding xylanolytic enzymes and includes regulation of three endoglucanase-encoding genes [16,17]. Although Van Peji *et al*. [16] reported endoglucanase expression in *A. niger*, they did not identify heterologous protein expression. Therefore, in this study, through nested PCR and function-driven screening, the endoglucanase gene *egl*B was identified using the genomic DNA of *A. niger*. *egl*B was expressed in *Picha pastoris*, and the recombinant enzyme was purified to characterize its properties.

## 2. Results and Discussion

### 2.1. Cloning, Sequencing, and Analysis of *egl*B

**Figure 1:**
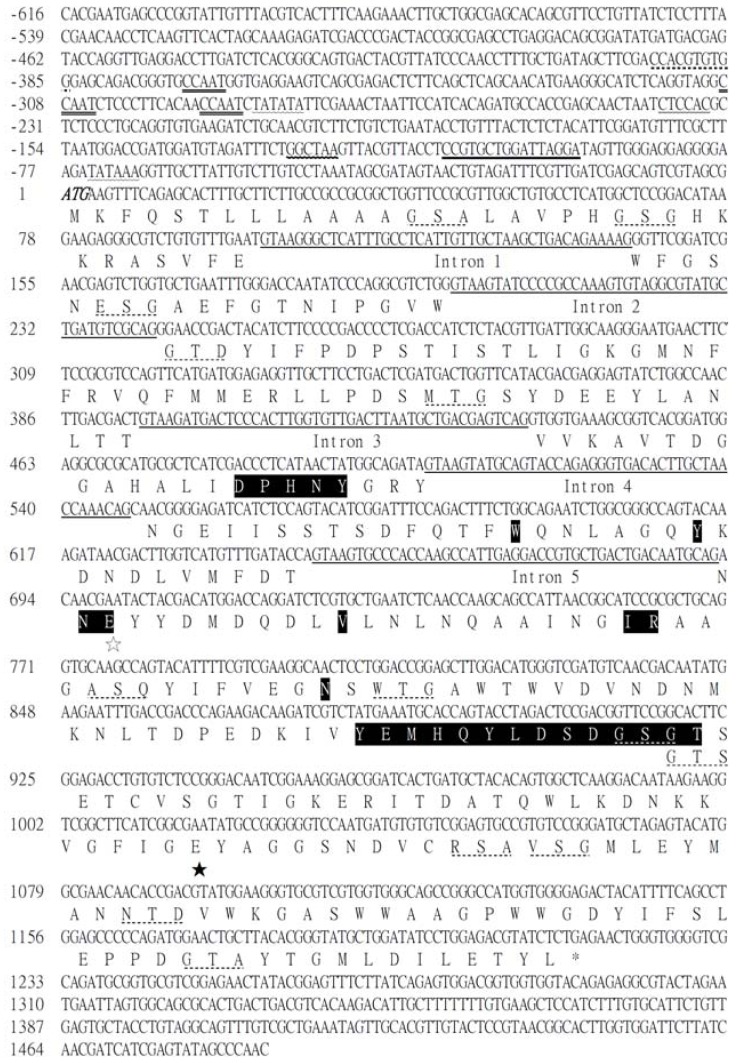
The nucleotide and deduced amino acid sequence of *egl*B. DNA sequences of intron are underlined. The start and stop cordons are shown bold italic. The putative CAAT box sequence is double-underlined. The putative TATA box sequence is wave-underlined. The putative XlnR binding sequence is bold wave-underlined. The putative CerA sequence is double wave-underlined. The putative CerE sequence is bold-underlined. The putative CCN_6_GG sequence is pointed. In the amino acid sequence, the solid bars indicate the regions conserving the catalytic nucleophile in glycosyl hydrolase family 5. The filled asterisk indicates the catalytic nucleophile. The hollow asterisk indicates the proton donor.

To sequence *egl*B, we used Primer Premier 5 software to design the primers. Two *egl*B sequences, *A. niger* (CBS 120.49) and *A. niger* CBS 513.88 with high similarity were used to designed primers, and seven sets of primers were designed for the nested PCR. The genomic DNA of *A. niger* BCRC 31494 was used as a template, and the expected fragments were purified and sequenced. After comparing the results of RT-PCR and nested PCR with DNAMAN software, the full-length sequences of *egl*B (1,217 bp) and cDNA (996 bp) were obtained (Figure 1, GenBank GQ292753). Five introns were identified. The lengths of the introns were 43, 43, 45, 43, and 47 bp. This gene followed the GT…AG exon principle. The deduced amino acid sequence of 332 amino acids exhibited high homology to those of other fungal enzymes, including 100% identity to endo-beta-1,4-glucanase B of *A. niger* CBS 513.88 (NCBI Reference Sequence: XP_001391969), 97% identity to the endoglucanase precursor of *A. niger* (GenBank™ accession AAG50051), 97% identity to endoglucanase C of *A. kawachii* (GenBank™ accession BAB62319), 79% identity to cellulose of *Neosartorya fischeri *NRRL 181 (NCBI Reference Sequence: XP_001257866), 77% identity to endo-beta-1,4-glucanase B of *A. oryzae* RIB40 (NCBI Reference Sequence: XP_001818463), and 76% identity to endo-1,4-beta-glucanase of *A. oryzae *(GenBank™ accession BAD72778). This gene was identified as EGLB. The cloned gene lacked the cellulose-binding domain sequence.

As shown in Figure 1, the 5′ end of the sequence (approximately 600 bp) included most of the regulatory sequences. In the upstream region, TATA-like sequences, which can be recognized by RNA polymerase II in eukaryotes, were present at positions −286 and −74. The CCAAT box sequences at positions −371, −309, and −292, are rather common in fungi and participate in Hap complex binding. A zinc binuclear cluster transcription factor, xylanolytic activator (XlnR), may be present at position −128. XlnR is homologous with Xyr1, which exists in all cellulolytic and xylanolytic enzymes [16]. However, XlnR only exists in some *Aspergillus* species. According to current research on Xyr1, sophorose is its most effective inducer. Some research indicated that the function of the two genes is almost similar, indicating that XlnR might also be induced by sophorose [19]. Upon induction, the enzyme activity can exceed the baseline activity by 2,500-fold. 

The carbon catabolite repression (CreA) binding site (5′-SYGGRG-3′) at position −239 is required for the mediation of carbon catabolite repression by EGLB. CreA can be classified into three types according to the mechanism of gene regulation. The most important type is translated into CreA protein, which catabolizes the carbon source. The second type is gene repression by the final product. The third group includes the repression of gene translation and gluconeogenic and glyoxylate cycle enzymes. CreA is very important in the regulation of metabolism achieved through a complex enzyme system and signal transduction [20].

A cellulose responsive element (CeRE) sequence is located from −110 to −95, and a CCN6GG sequence is located at position −394 (Figure 1). CeRE (CCGTGCTGGATTAGGA) is located upstream of *egl*B. Mutations at −109 of *egl*B ablate the function of CeRE [21]. Sequences similar to cellulolytic enzyme promoters have been found in *Aspergillus *species and other species, such as *A. nidulans* and *A. oryzae*, but the detailed mechanism has not yet been thoroughly studied [21]. The CCN6GG sequence upstream of *egl*B was inverted and repeated in the *egl*B of *A. nidulans*. The present study for the detailed function has not been clarified, but deletion or mutation of this sequence can indeed reduce the expression of the *egl*B gene [21].

The amino acid sequence of the purified EGLB was analyzed using OGPET [Prediction of *O*-GalNAc (mucin-type) glycosylation sites in eukaryotic (non-protozoan) proteins] software, and 13 possible *egl*B glycosylation positions were found. From the result of RPS-BLAST alignment, *egl*B belongs to glycosidase family 5 of the cellulase superfamily. The proton donor of Glu160 and catalytic nucleophile of Glu266 are consistent with the characteristics of family 5 [22]. Cellulose is a macromolecular compound; it cannot be directly transported into the cell for decomposition. Therefore, the release of extracellular enzymes is necessary. Thus, the signal sequence of *egl*B was also determined using Signal P 3.0. The result suggested that amino acid residues 1–18 comprise a signal peptide, and the cleavage site was between residues 18 and 19 from the first methionine found in the amino acid sequence. The highly conserved sequence was followed by catalytic nucleophiles, which may be glycosylation sites, and several possible glycosylation sequences were found in this fragment. Another possibility is that these highly conserved sequences were the glycosyl residues of family 5. The glycosidic bond cleavage reaction, including the proton donor, catalytic nucleophile, and five other conserved residues of family 5 are involved belonging to retaining mechanism [22,23].

### 2.2. Expression, Purification, and Deglycosylation of EGLB

**Figure 2:**
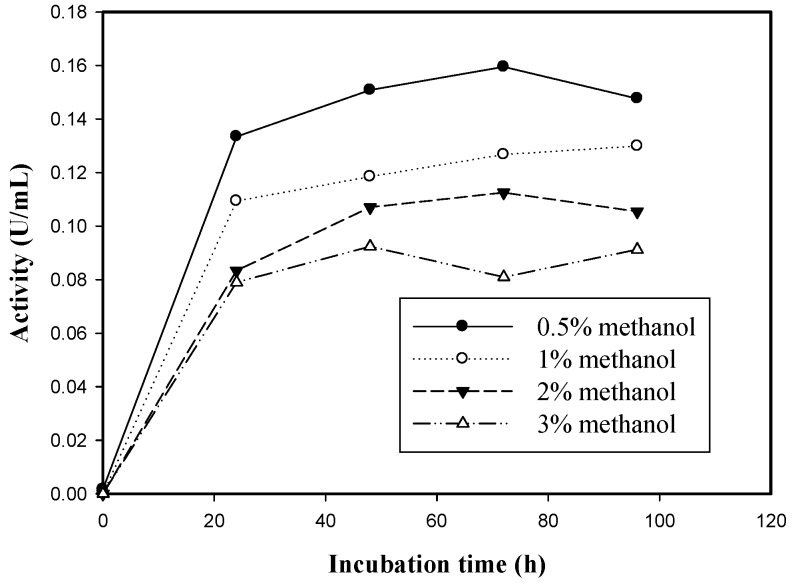
Strain 31 was induced with different concentrations (0.5, 1, 2, and 3%) of methanol.

A recombinant EGLB protein was successfully expressed in *P. pastoris* and secreted into the medium. After induction with methanol, the carboxymethyl cellulose (CMC) hydrolysis activity of the 70 transformants was analyzed, and 10 transformants with high EGLB activity were selected. Strain 31 had the highest endoglucanase activity, and it was used for subsequent experiments. In our laboratory, Strain 31 was induced with different concentrations (0.5, 1, 2, and 3%) of methanol, and induction with 0.5% methanol yielded the highest enzyme activity (1.35 U/mg, Figure 2). Thus, 0.5% methanol was used for large-scale expression. Kanokratana *et al*. [24] revealed that higher methanol concentrations can result in a higher protein yield. In this study, contrary results were obtained. A lower concentration of methanol (0.5%) resulted in the highest enzyme activity. This may be because Mut^−^ strains of *P. pastoris* grow poorly in the presence of methanol, and heterogenous protein expression was inhibited [25].

**Figure 3:**
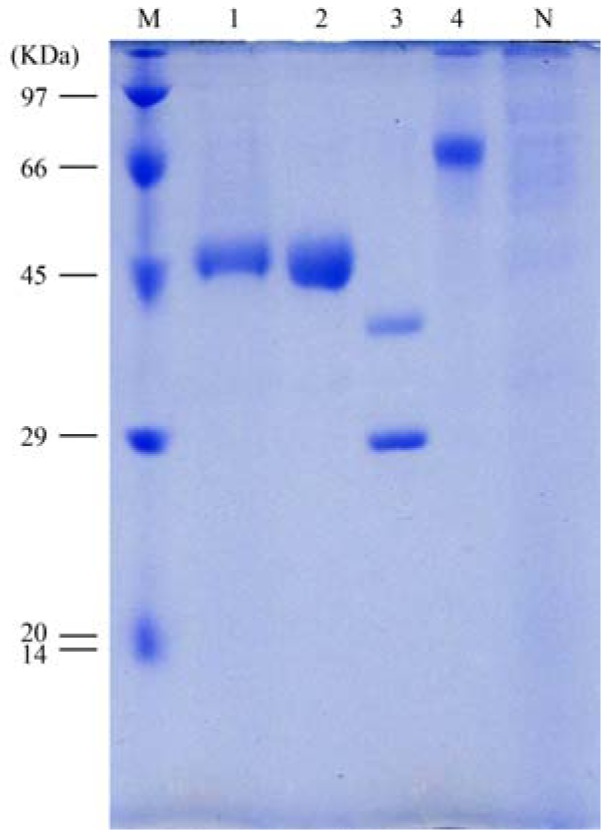
SDS-PAGE of the purified recombinant EGLB. SDS-PAGE of EGLB purified from *P. pastoris* GS115-31. Lane M, molecular marker; Lane 1, culture supernatant (4 days); Lane 2, purified EGLB; Lane 3, Endo H-treated EGLB; Lane 4, BSA; Lane N, cultured supernatant without induction.

The culture supernatant was collected, concentrated, and purified by His-bind resin chromatography. SDS-PAGE data revealed that the recombinant EGLB was the major protein in the culture supernatant, with a clear band of approximately 51 kDa in size (Figure 3). The molecular weight of the band was higher than the calculated molecular weight of the protein (46.3 kDa). The purified recombinant EGLB was hydrolyzed by endoglycosidase H, and the hydrolysate was analyzed by SDS-PAGE. The bare protein was recognized as a single band at 43.6 kDa (Figure 3). The band of approximately 30 kDa corresponds to the Endo H enzyme in the text. This suggested that the recombinant EGLB was an N-linked glycosyl protein. The recombinant EGLB yielded a clear band with a pI of 4.75 according to PhastGel IEF analysis. The purified protein was digested with trypsin, and the resulting peptides were analyzed by LC-MASS. The peptides GMNFFR, VQFMMER, ITDATQWLK, ITDATQWLKDNK, ITDATQWLKDNKK, VGFIGEYAGGSNDVCR, and AVTDG GAHALIDPHNYGR completely corresponded to the amino acid sequence of EGLB, and no peptides from other proteins were detected, confirming the purity of the band and the identity of EGLB. The specific activity of purified EGLB was 138 U/mg in a shaker flask culture after induction with 0.5% methanol for 4 days. 

### 2.3. Optimal pH and Temperature of the Recombinant EGLB in *Pichia pastoris*

**Figure 4:**
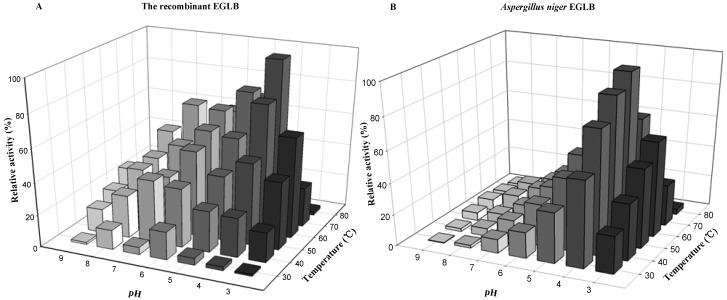
Optimum temperature and pH of the purified EGLB.The optimal temperature and pH for EGLB were determined by incubating the purified enzyme in different buffers (50 mM, pH 3.0 to 9.0) and then exposing them to different temperatures (from 30 °C to 80 °C) with CMC-Na (0.5%, w/v) as substrate for 30 min. Each enzymatic reaction was determined by the standard enzyme method (Section 3.11). Each value represents the mean of triplicate measurements, and varied from the mean by not more than 10%. The used buffers were 50 mM citrate (pH 3.0–5.0), 50 mM sodium acetate/acetic acid (pH 4.0–6.0), Na_2_HPO_4_/NaH_2_PO_4_ (pH 5.0–8.0), and Tris-HCl (pH 8.0–9.0).

The recombinant enzyme EGLB was more stable than the wild-type enzyme. The wild-type enzyme was purified according Vidmar *et al.*’s method [26]. The relative activity of the recombinant EGLB at pH 5–9 was significantly higher than that of the wild-type (Figure 4A,B). The recombinant enzyme displayed broad pH stability; when incubated at 70 °C in the pH range of 4.0 to 8.0, the enzyme activity remained higher than 50%. The recombinant enzyme EGLB had an optimal temperature of 70 °C, which was higher than that of the wild-type enzyme (60 °C). Its high optimal reaction temperature and reactivity at a broad pH range make the recombinant enzyme applicable in the bioresource utilization industry. Industrial application requires an enzyme with thermal stability and reactivity at a broad pH range. In a previous papers, endoglucanases with high optimal temperatures were reported, such as the wild-type endoglucanase B of *A. niger* (60 °C) [26], the thermotolerant endoglucanase of *Syncephalastrum racemosum* (70 °C) [27], and the endo-β-1,4-glucanase of *Bacillus subtilis* BME-15 (50 °C) [28]. The recombinant enzyme EGLB has an optimal pH of 4.0. 

**Figure 5:**
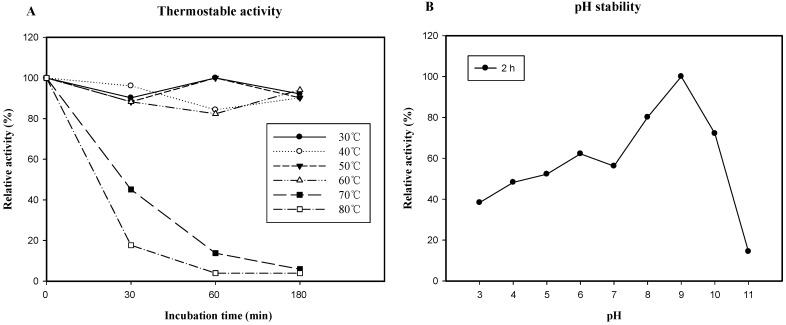
Effect of temperature and pH on the activity of recombinant endoglucanase B.(**A**) Thermostability of recombinant EGLB. The recombinant enzyme was pre-incubated at various temperatures in 50 mM Na-acetate buffer (pH 5.0) in the absence of substrate. Aliquots were taken at specific time points for the measurement of residual activity by the standard assay method (Section 3.11). (**B**). The pH stability of recombinant EGLB was determined by pre-incubating the enzyme at 37 °C for 2 h in different buffers (50 mM, pH 3.0–11.0). After adjusting each enzyme solution to pH 4.0, the remaining enzyme activity was determined by the standard assay method. Each value represents the mean of triplicate measurements, and varied from the mean by not more than 10%.

The thermal stability of EGLB is presented in Figure 5A; approximately 90% of the purified EGLB activity remained when the enzyme was incubated at temperatures less than 60 °C for 30 min. Even at 70 °C, half of the enzyme activity remained. The enzyme exhibited a half-life of 30 min at 70 °C and 25 min at 80 °C. Vidmar *et al*. [26] reported that the wild-type endoglucanase B of *A. niger* displayed thermal stability (up to 50 °C) in acidic conditions. In this study, the thermal stability of EGLB at temperatures as high as 60 °C at pH 5.0 was higher than that reported by Vidmar *et al*. [26]. The thermal stability of *endo*-β-1,4-glucanase B from *A. oryzae* KBN616 was stable up to 50 °C and became inactivate rather sharply above 55 °C [3]. The EglA from *A. niger* ATCC10574 was stable at temperature between 30 to 50 °C for 30 min [15] and the EglC from *A. niger* CBS120.49 was stable at 30 °C for 148 h [17]. EGLB in this study had better thermal stability at higher temperature (60 °C) than the above mentioned enzymes. However, the ENG1 from *A. niger* IFO31125 showed thermal stable at 70 °C for 1 h [18] which is similar to EGLB. These results indicated that EGLB produced in yeast is stable at high temperature. Therefore, the recombinant enzyme has potential for use in higher temperature industrial fermentation applications.

The pH stability of EGLB is shown in Figure 5B. The enzyme was stable at alkaline pH. Most fungal endoglucanases were stable in acidic pH, for examples *endo*-β-1,4-glucanase B from *A. oryzae* KBN616 was stable in pH 3 to 7 [3], the EglA from *A. niger* ATCC10574 was stable between pH 2 to 7 [15], and the EglC from *A. niger* CBS120.49 was stable at pH 3.5 to 5 [17]. The EGLB expressed from *A. niger* in this study was stable at pH 3 to 10, and very stable at alkaline region (Figure 5B). This data was consistent with that the endoglucanase from *Volvariella volvacea* was an alkaline pH stability enzyme from fungi [29]. Alkaline cellulases have new industrial applications as laundry detergent additives [4]. Endoglucanases from *Bacillus* spp. always display high thermal stability and alkaline pH stability [30]. EGLB has a high optimal temperature of 70 °C, displays thermal stability at 60 °C, and is stable at pH 9.0. These properties indicate the EGLB can be used as an additive in laundry detergent.

### 2.4. Substrate Specificity and Kinetic Parameters of EGLB

The substrate specificity and kinetic parameters of EGLB were measured by releasing reducing sugars from several substrates with various linkages (Table 1). The highest activity was detected for barley β-glucan, followed by sodium carboxymethyl cellulose (CMC-Na). Laminarin, xylan from beechwood, chitin, and salicin were slightly degraded by EGLB. The Michaelis constant (*K_m_*) and maximum velocity (*V_max_*) of recombinant EGLB for barley β-glucan were 53 mg/mL and 134 U/min/mg, respectively, compared with 32 mg/mL and 4.68 U/min/mg, respectively, for CMC-Na. Kinetic analysis revealed that EGLB has a greater affinity for barley β-glucan than for CMC-Na. The result confirmed that EGLB is a β-1,3-1,4-glucanase. Recently, an endoglucanase from the acidophilic fungus *Bispora *sp., MEY-1, was also reported as an *endo*-β-1,3-1,4-glucanase [31].

**Table 1:** Substrate specificity and kinetic parameters of the purified recombinant EGLB.

Substrate	Main linkage (monomer)	Specific activity (U/mg)	*K_m_*(mg/mL)	*V_max_* (U/min/mg)
β-D-glucan from barley	1,3-1,4-β-(glucose)	405	53.0	32.4
locust bean gum	backbone of (1-4) β-D-mannopyranosyl units branches of (1-6)-linked α-D-galactopyranosyl units	171		
cellobiose	1,4-β-(glucose)	78		
CMC-Na	1,4-β-(glucose)	68	135	4.7
Laminarin	1,3-β-(glucose)	26		
xylan from beechwood	1,4-β-(xylose)	19		
Chitin	1,4-β-(glucosamine)	10		
Salicin	β-(glucose)	6		
Avicel	1,4-β-(glucose)	0		
*p*-nitrophyl-α-D-glucopyranoside		0		
*p*-nitrophyl-β-D-glucopyranoside		0		

### 2.5. Effects of Metal Ions and Chemicals on EGLB Activity

The effects of metal ions and various compounds (10 mM) on EGLB activity are shown in Table 2. The enzyme activity was enhanced in the presence of Co^2+^, Triton X-100, and Tween 80 by 186, 201, and 205%, respectively. The enhancement of endoglucanase activity by cobalt ions was previously reported in bacteria [30], and also observed in fungi, the EglA from *A. niger* ATCC10574 (160%) [15] and the EGVIII from *Trichoderma virde* (130%) [32]. Surfactants are protein-denaturing agents. However, in this study, Tween 80 and Triton X-100 enhanced EGLB activity by approximately 200%. Many studies have reported that surfactants can improve the digestibility of several types of enzymes [33,34]. Zheng *et al*. [33] divided the mechanisms into three primary categories: (1) stabilizing the enzyme by reducing thermal and/or mechanical shear forces; (2) changing the ultrastructure of the substrate, making the cellulose more accessible to enzymes; and (3) affecting the enzyme-substrate interaction. To our knowledge, this is the first report indicating that EGLB from *A. niger* can be activated by Triton X-100 and Tween 80. However, the enzyme was strongly inhibited by SDS. Partial inhibition (<20%) was observed in the presence of certain metal ions, including Mg^2+^, Zn^2+^ and Ca^2+^, whereas the enzyme activity was not affected by Cu^2+^ and EDTA.

**Table 2:** Effects of metal ions, surfactants and EDTA on the purified recombinant EGLB activity.

Compound added	Conc. (mM)	Relative acitivity(%)
None		100
CdCl*_2_*	1	102
	5	88
	10	79
FeCl*_2_*	1	84
	5	85
	10	86
MgCl*_2_*	1	87
	5	84
	10	77
CaCl*_2_*	1	88
	5	85
	10	75
CoCl*_2_*	1	141
	5	175
	10	186
ZnCl*_2_*	1	85
	5	81
	10	68
CuCl*_2_*	1	100
	5	104
	10	117
Triton X-100	10	201
Tween 80	10	205
SDS	1	80
	5	78
	10	1
EDTA	1	92
	5	93
	10	110

The purified enzyme was incubated with the added compound for 10 min prior to addition of 0.5% (wt/v) CMC-Na in Na/acetic buffer (pH 5.0) for 30 min at 40 °C. Values represent the relative activity expressed as a percentage of the activity observed in the absence of compound. Values shown are the mean of duplicate experiments, and the variation about the mean was <5%.

## 3. Experimental

### 3.1. Strains, Plasmids, and Media

*Aspergillus niger* BCRC 31494 and *Escherichia coli* DH5α were purchased from the Bioresource Collection and Research Center (BCRC), Food Industry Research and Development Institute (Hsein-chu, Taiwan). *Pichia pastoris* GS115 was obtained from Invitrogen (Carlsbad, CA, USA). PDA slant medium (1.8% Bacto-agar and 2.4% Potato dextrose) was used to culture the fungal spores at 30 °C for 3 days. After inoculating spores (1 × 10^7^) into 250 mL of minimal medium (0.05% (NH_4_)_2_SO_4_·3H_2_O, 0.02% KH_2_PO_4_, 0.02% MgSO_4_, 0.01% CaCl_2_·H_2_O, 0.0001% FeSO_4_·6H_2_O, 0.0001% ZnSO_4_·7H_2_O, 2 mM citric acid, and 1% bran, pH 3.5) at 37 °C and 150 rpm for 3 days, the mycelia were harvested and used for DNA and RNA isolation. *Pichia pastoris* GS115 was cultured in YPD medium (1% yeast extract, 2% peptone, and 2% dextrose) at 30 °C and 200 rpm. *E. coli* DH5α was grown in LB medium (1% tryptone, 0.5% yeast extract, and 1% NaCl, pH 7.5) or low salt LB medium (1% tryptone, 0.5% yeast extract, and 0.5% NaCl, pH 7.5) at 37 °C and 200 rpm for DNA manipulation and expression. The plasmid pPICZαC was used as an expression vector and purchased from Invitrogen.

### 3.2. Total RNA, Genomic DNA, and Plasmid DNA Isolation

To isolate total RNA, *A. niger* BCRC 31494 was grown in 250 mL of minimal medium at 37 °C and 150 rpm for 3 days. The mycelia were harvested and then ground into a fine powder in liquid nitrogen with a mortar and pestle. The mycelia were then lysed, and total RNA was isolated using TRIzol^®^ and an RNA purification kit (Invitrogen) according to the manufacturer’s protocol. Genomic DNA was isolated from *A. niger* BCRC 31494 using the Plant Genomic DNA Mini Kit (Geneaid, New Taipei, Taiwan) according to the manufacturer’s protocol. Plasmid DNA isolated from *E. coli* DH5α was extracted using the Mini M™ Plasmid DNA Extraction System (Viogene, New Taipei, Taiwan) according to the manufacturer’s protocol.

### 3.3. Construction of the *egl*B Library

Genomic DNA was isolated from mycelia cultured for 3 days. Degenerate primers were designed according to two gene sequences of *A. niger* (NCBI GenBank accession Nos. AJ224452 and XM_001391932). Nested PCR, using genomic DNA as a template, was performed using TaKaLa LA Taq (TaKaRa Shuzo, Kyoto, Japan). The PCR reaction product, consisting of partial sequences of the putative *egl*B, was cloned into yT & A vectors by using a yT & A cloning kit (Yeastern Biotech., Taipei, Taiwan) and sequenced. This sequence was then used to design another set of internal primers against the putative *egl*B for the next round of nested PCR. Ligation-anchored PCR (LA PCR) was performed using an LA PCR in vitro cloning kit (TaKaRa Shuzo) according to the manufacturer’s protocol. Sequence analysis was performed using DNAMAN software, and sequencing was performed by the TriLigo Company (New Taipei, Taiwan).

### 3.4. RT-PCR of a cDNA Encoding *egl*B

Total RNA was isolated from mycelia cultured for 3 days. The full-length cDNA was obtained using a ReverTra Ace kit (Toyobo, Osaka, Japan) according to the manufacturer’s protocol. Next, the *egl*B cDNA sequence was amplified using primers *egl*B-F (5′-ATGAAGTTTCAGAGCACTTT-3′) and *egl*B-R (5′-TCAGAGATACGTCTCCAGGA-3′) with the Advantage 2 PCR Enzyme System (BD Biosciences, San Jose, CA, USA). The amplified DNA fragments were cloned into yT & A vectors (yT & A-*egl*B) by using the yT & A cloning kit and transformed into *E. coli* DH5α, and colonies were selected by blue-white selection. Sequence analysis was performed using DNAMAN software, and sequencing was performed by the TriLigo Company.

### 3.5. Construction of the *egl*B Expression Vector

To construct the *egl*B expression vector, two primers, P1 (5′-CCCC*GAATTC*TGTGCCTCATGGCTCCGGACA-3′), with an added *Eco*RI site, and P2 (5′-GC*TCTAGA*TTGAGATACGTCTCCAGGA-3′), with an added *Xba*I site, were designed. The plasmid yT & A-*egl*B was used as the template, and TaKaLa LA Taq (TaKaRa Shuzo) was employed. The following PCR cycling conditions were used: 94 °C for 1 min; 30 cycles of 94 °C for 30 s, 58 °C for 1 min, and 72 °C for 3 min; and 72 °C for 10 min. DNA fragments amplified by PCR were cut by *Eco*RI and *Xba*I and then purified for ligation. The synthesized *egl*B cDNA was ligated to an *Eco*RI- and *Xba*I-cut pPICZαC vector, and the resulting plasmid was designated pPICZαC-*egl*B. Transformation of *E. coli* DH5α was performed using aYE607 kit (Yeastern Biotech).

### 3.6. P. pastoris Transformation

Transformation of *P. pastoris *GS115 was performed according to Wu and Letchworth’s method [35]. The transformants were transferred to YPDS plates (YPD medium containing 1 M sorbitol and 2% agar), and YPDS containing different concentrations of zeocin (500, 1,000, and 2,000 μg/mL) was used as the selection medium.

### 3.7. P. pastoris Induction and Expression

A small-scale culture was used for selecting strains with high EGLB activity. A single colony was picked from the purified *Pichia* colonies, inoculated into YPDZ medium (YPD medium containing 100 μg/mL zeocin), and then grown in a shaking incubator at 200 rpm and 30 °C for 24 h. The culture was inoculated in 3 mL of BMGY medium (1% yeast extract, 2% peptone, 100 mM potassium phosphate buffer, pH 6.0, 1.34% yeast nitrogen base, 4 × 10^−5^% biotin, and 1% glycerol) and grown in a shaking incubator at 200 rpm and 30 °C for 24 h. The cell pellets were harvested, resuspended in 3 mL of BMMY medium (BMGY medium without 1% glycerol but with 0.5% methanol) to induce expression, and incubated at 30 °C to continue growth. Sterilized pure methanol was added at a final concentration of 1% every 24 h to maintain induction for 4 days. The expressed protein and the endoglucanase of the supernatants and cell pellets were analyzed by SDS-PAGE and stained with Coomassie Blue.

Mass production of the expressed enzyme was performed by large-scale culturing. The most efficiently expressed single *Pichia* colony, No. 31, was inoculated in 25 mL of BMGY medium. The cell pellet was then harvested and resuspended in BMMY medium to induce enzyme expression. Sterilized pure methanol was added at a final concentration of 0.5, 1, 2, or 3% every 24 h to maintain induction for 4 days. The expressed protein and the endoglucanase of the supernatant and cell pellet were analyzed by SDS-PAGE and stained with Coomassie Blue. 

### 3.8. Purification of the Recombinant EGLB from P. pastoris No. 31

After cultivation, the supernatant was collected by centrifugation at 3,000 × *g* for 5 min. EGLB was purified by His-bind resin chromatography (Novagen, Madison, WI, USA) according to the manufacturer’s manual. The purified EGLB was pooled, concentrated (Millipore ultrafiltration membrane with a 10-kDa cutoff), and dialyzed with PBS (pH 7.0).

*Deglycosylation analysis*: The purified recombinant EGLB (1 μg) was deglycosylated with 250 U of endoglycosidase H for 1 h at 37 °C according to the manufacturer’s instructions (New England Biolabs, Beverly, MA, USA). The deglycosylated and the untreated EGLB were analyzed by SDS-PAGE.

### 3.9. pI Analysis

The pI of the purified recombinant EGLB was analyzed by PhastSystem PhastGel IEF (GE Healthcare Life Science, Piscataway, NJ, USA). The PhastGel IEF gel was silver-stained.

### 3.10. LC-MASS Assay

The purified enzyme was analyzed by SDS-PAGE according to Laemmli’s method [36], and the protein spot in question was picked up using a pipette tip. The protein spot was treated with a reducing agent (dithiolthreitol) to reduce disulfide bonds and discolored with acetonitrile. Trypsin was added to the spot to digest the enzyme in the gel. The spot was then assayed using LTQ FT ULTRA (Thermo Scientific, San Jose, CA, USA).

### 3.11. Assay of Endoglucanase Activity

Endoglucanase activity was determined by measuring the increase in the amount of reducing sugar released from CMC-Na using the dinitrosalicylic acid method [37]. Sodium acetate-acetic acid buffer (50 mM, pH 5) containing 0.5% (w/v) CMC-Na was used. One unit of enzyme activity was defined as the amount of protein that produced 1 μg of product per mL per min from 0.5% CMC-Na at 40 °C for 30 min. Each reaction and its control were run in triplicate.

### 3.12. Optimal Temperature and pH Determination

The optimal temperature and pH of EGLB were determined at different temperatures (from 30 to 80 °C) and in different buffers (pH 3.0–9.0) for 30 min. The buffers used were as follows: 50 mM citrate for pH 3.0–5.0, 50 mM sodium acetate/acetic acid for pH 4.0–6.0, Na_2_HPO_4_/NaH_2_PO_4_ for pH 5.0–8.0, and Tris-HCl for pH 8.0–9.0. EGLB activity was measured as described previously. 

The thermal stability of the purified recombinant enzyme was determined by assessing the residual enzyme activity under standard conditions after incubating the enzyme at different temperatures (30–80 °C) in the absence of substrate for certain periods of time (30, 60, and 180 min). 

The pH stability of EGLB was determined by measuring the residual activity under standard conditions (pH 5.0, 40 °C for 30 min) after incubating the enzyme at 37 °C in different buffers (pH 3.0–9.0, described previously) for 2 h in the absence of substrate.

*Substrate specificity*: The substrate specificity of the recombinant EGLB was determined using CMC-Na, avicel, beechwood xylan, laminarin, barley β-glucan, salicin, chitin, locust bean gum, cellobiose, *p*-nitrophenyl-α-D-glucopyranoside and *p*-nitrophenyl-β-D-glucopyranoside as substrates at concentrations of 0.5% (w/v). The amount of reducing sugar released was determined by the DNS method described previously.

### 3.13. Determination of Kinetic Parameters

The *K_m_* and *V_max_* of EGLB were determined via incubation in 50 mM Na-acetate buffer (pH 5.0) at 40 °C with CMC-Na or barley β-glucan at concentrations ranging from 0.001 to 0.1 g/mL. The *K_m_* and *V_max_* values were determined from Lineweaver-Burk plots by using standard linear regression techniques.

### 3.14. Effect of Metal Ions and Chemicals on Enzyme Activity

The effects of metal ions and regents at a concentration 10 mM on EGLB activity were determined by preincubating the enzyme with the individual reagents in 50 mM Na-acetate buffer, pH 5.0 at room temperature for 10 min. The residual activity was then determined. The activity assayed in the absence of metal ions or regents was recorded as 100%.

## 4. Conclusions

In summary, an EGLB from *Aspergillus niger* (BCRC31494) has been successfully cloned and sequenced. The cloned gene was expressed in *Pichia pastoris*, the extracellular EGLB was purified, and the properties of the enzyme were characterized. Specifically, the activity of the enzyme was enhanced in the presence of Co^2+^, Triton X-100, and Tween 80. The recombinant EGLB exhibited high thermotolerance and stability in alkaline conditions, indicating that the enzyme can be effectively used in bioethanol production and in laundry, textile, denim staining, and paper recycling applications in industry.
